# Mixed U‐Net: Segmentation of focal liver lesions using a hybrid 2D and 3D model

**DOI:** 10.1002/acm2.70408

**Published:** 2025-12-28

**Authors:** Feifei Gao, Zheming Hu, Jiajun Xian, Wei Lu

**Affiliations:** ^1^ Department of Radiology West China Hospital Sichuan University Chengdu China; ^2^ School of Computer Science and Engineering University of Electronic Science and Technology of China Chengdu Sichuan China; ^3^ Yangtze Delta Region Institute (Quzhou) University of Electronic Science and Technology of China Quzhou Zhejiang China; ^4^ Quzhou Affiliated Hospital of Wenzhou Medical University Quzhou People's Hospital Quzhou Zhejiang China

**Keywords:** 2.5D segmentation, computed tomography, convolutional neural network, liver tumor segmentation, mixed 2D and 3D convolutional network, U‐Net

## Abstract

**Background:**

Accurate segmentation of liver tumors on contrast‐enhanced CT is essential for clinical decision‐making, but remains challenging due to irregular tumor boundaries and the difficulty of distinguishing lesions from blood vessels and bile ducts. Although 3D convolutional networks effectively capture inter‐slice contextual information, they are computationally intensive and memory‐demanding. In contrast, 2D networks are efficient but limited by their inability to model volumetric context, often resulting in discontinuous or inaccurate segmentations. 2.5D approaches that stack adjacent slices offer a compromise but suffer from early fusion of information that weakens spatial discrimination.

**Purpose:**

We propose Mixed U‐Net, a hybrid segmentation architecture designed to extract fine‐grained z‐axis features while maintaining low computational cost.

**Methods:**

We embed a small number of 3D convolutional layers into a 2D convolutional U‐Net using residual and skip connections. This allows the 3D convolutions to perform fine‐grained spatial feature extraction at multiple depths in the network, effectively simulating 3D segmentation within a lightweight framework. Mixed U‐Net was trained and evaluated on a dataset of 532 liver tumor cases from Hospital A, and externally validated on 45 cases from Hospital B.

**Results:**

Mixed U‐Net achieved a Dice score of 81.54% (95% CI: 81.45%–81.62%) on the internal test set, outperforming multiple 2D, 3D, and 2.5D baselines. On the external dataset, it maintained strong performance with a Dice of 78.92%, exceeding the baseline by 4.67%, demonstrating superior generalization.

**Conclusions:**

By integrating 3D feature extraction into a primarily 2D architecture, Mixed U‐Net balances contextual accuracy with computational efficiency, making it well‐suited for clinically applicable liver tumor segmentation.

## 1 INTRODUCTION

In 2020, primary liver cancer was the sixth most common cancer worldwide and the third leading cause of cancer death, with approximately 906,000 new cases and 830,000 deaths.^1^ Hepatocellular carcinoma (HCC) accounts for 75% to 85% of all cases of primary liver cancer, while intrahepatic cholangiocarcinoma (ICC) accounts for 10% to 15%, and there are also other rare types.^1^ Additionally, secondary liver cancer is more common than primary liver cancer.^2^ Medical image segmentation plays a crucial role in the diagnosis and treatment of liver cancer. Accurate segmentation of liver tumors is indispensable in diagnosing liver cancer, monitoring disease progression, locating lesions, calculating remnant liver volume (RLV), devising treatment plans, and evaluating the subsequent effects of treatment.^3^, ^4^, ^5^ The traditional manual segmentation process is laborious and time‐consuming, and the quality of segmentation depends on the reliability of the observer. In contrast, deep learning algorithms have achieved significant success in the field of medical imaging.^6^, ^7^ Specifically, deep learning‐based automatic medical image segmentation—which simulates experienced clinicians to perform lesion segmentation—is an efficient and reliable tool for analyzing disease progression and treatment efficacy.^6^, ^8^

In clinical practice, accurate liver tumor segmentation on contrast‐enhanced CT is challenged by several factors. First, tumor boundaries often appear ill‐defined against adjacent liver parenchyma, making it difficult to delineate lesions. Second, portal and hepatic vessels, artifacts, and dilated bile ducts often display enhancement patterns or intensities similar to tumors, resulting in frequent false positives. Third, liver lesions vary widely in size, shape, texture, and number across different patients and tumor types, further complicating reliable detection. These challenges highlight the need to use three‐dimensional context from adjacent CT slices, which can improve boundary clarity, reduce misclassification of vessels or bile ducts, and better handle varied lesion characteristics.

Deep learning‐based automatic medical image segmentation typically involves three methods.^9^ The first, 2D segmentation, uses networks like FCN,^10^ U‐Net,^11^ U‐Net++,^12^ and DeepLabV3+.^13^ Furthermore, models based on vision transformers^14^ such as SegFormer^15^ have also demonstrated excellent performance in image segmentation. However, by processing each slice independently, these 2D models are computationally efficient but ignore z‐axis context. As a result, they produce discontinuous masks between adjacent slices,^16^ and often mistake enhanced vessels or dilated bile ducts for tumors, since those structures can appear similarly bright on single slices. Such errors undermine the volumetric consistency and anatomical accuracy required for reliable clinical decision‐making. Another approach is 3D segmentation, which can address the limitations of 2D segmentation. The primary architectural difference between 3D and 2D convolutional networks lies in the extension of spatial operations to the volumetric domain. For instance, the 3D U‐Net^17^ extends the original architecture by employing 3D convolutions, 3D pooling, and 3D deconvolution for upsampling operations to process volumetric data, while maintaining the original architecture of the U‐Net network. The increased input dimensions and convolutional kernel dimensions of 3D models imply significantly higher computational costs and require more expensive GPUs. Moreover, due to limitations in GPU memory size, a common strategy is to use randomly cropped blocks of CT volume data as network input. While ensuring certain z‐axis features, there is a loss of overall spatial features along the x and y axes. The 2.5D segmentation method is employed to overcome the limitations of both 2D and 3D segmentation, achieving z‐axis feature extraction while reducing computational costs. The method stacks adjacent slices along the channel dimension as input for a 2D network. This approach allows for the extraction of z‐axis information from adjacent slices while retaining the efficiency and effectiveness of 2D networks. Yet, it has drawbacks in the extraction and integration of information from individual slices. The first convolutional layer merges all slice information into a single channel, thereby causing information to be conflated. Regarding the sequence‐related spatial information of slices, even if slices are stacked in a shuffled manner as input to the network with the context‐related information disrupted, this does not significantly impact the network's performance. Yu et al. also argue that this early fusion leads to information loss and poorer performance as more slices are stacked.^18^

In light of the above limitations of existing 2D, 3D, and 2.5D segmentation methods, this study aims to develop a computationally efficient yet context‐aware model for liver tumor segmentation. To balance the lightweight computational cost of 2D networks with more effective extraction of z‐axis features, we propose Mixed U‐Net—a hybrid architecture that integrates a small number of 3D convolutional layers into a 2D U‐Net framework via residual and skip connections. This design enhances volumetric contextual awareness while preserving efficiency, enabling fine‐grained inter‐slice feature learning without significantly increasing the number of parameters.

## 2 MATERIALS AND METHODS

### 2.1 Dataset development

The local institutional review board granted the ethics committee's approval, and this retrospective study was exempt from the requirement for informed consent. When constructing our dataset for experimentation, CT screening images of internal dataset taken between June 2012 and December 2022 at Hospital A were retrospectively selected. We collected CT data of 603 liver cancer patients, including HCC, ICC, and metastatic liver cancer (MET). All images were acquired during the portal venous phase. This phase is clinically preferred for liver lesion assessment because it provides strong contrast between the enhanced liver parenchyma and hypodense tumors,^19^ which is crucial for achieving accurate segmentation. The scans were performed using Siemens and Philips CT scanners. The images had a slice plane resolution of 512 × 512 pixels, and a slice thickness of either 2 or 5 mm.

**FIGURE 1:**
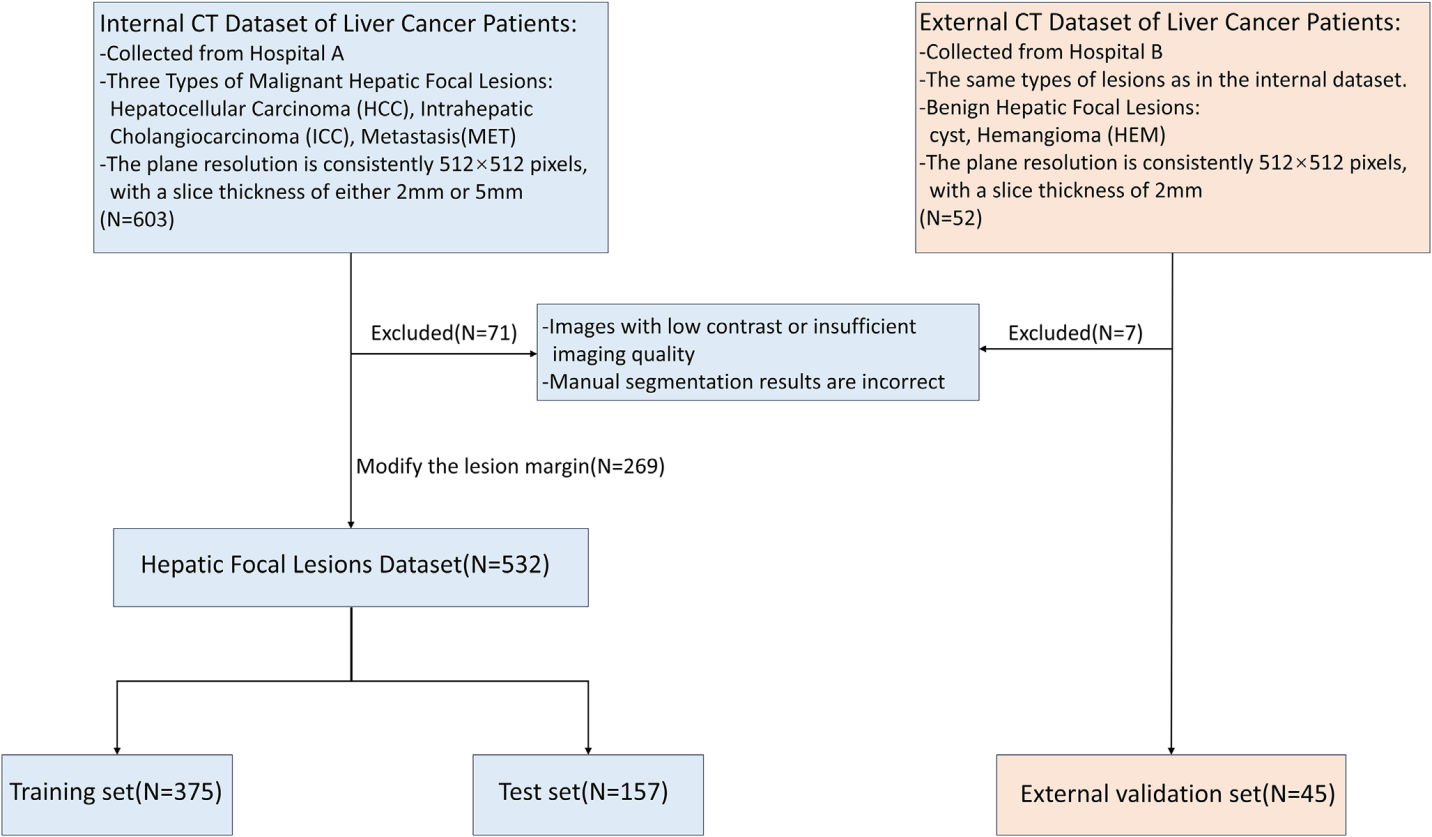
Dataset development flow chart.

This was a retrospective study where all patients met specific inclusion criteria to ensure the scientific validity and reliability of the experiment: (1) Age requirement: All patients must be over 18 years old to ensure that the study subjects have the physiological characteristics of adults. (2) Exclusion of surgical and treatment history: Patients must not have a history of liver resection, arterial chemotherapy, or radiofrequency ablation before undergoing CT examination to exclude the potential influence of these factors on the experimental results. (3) Nodule confirmation requirement: All nodules included in the study must be confirmed by pathology or have undergone follow‐up with at least two imaging modalities for 6 months to ensure the reliability and stability of the nodules. The region of interest for the lesions was manually delineated by radiologists. Two radiologists independently delineated the lesions under guidelines mandating that segmentations fully cover the target lesion with anatomically reasonable boundaries suitable for clinical application. All annotations were cross‐verified for consistency. After manual annotation, the image quality and segmentation results were reviewed, according to the exclusion criteria: (1) images with low contrast or low imaging quality, (2) images with errors in manual segmentation results. Subsequently, images with insufficiently accurate manual segmentation of lesion edges were modified. A total of 71 patients were excluded, and 269 cases underwent manual correction, which included refining ambiguous boundaries, removing non‐target annotations, and adding missed lesion areas. Finally, 532 patients were included. All CT images were set as window level of 50 HU and window width of 400 HU. The patient data was randomly split into training and testing sets in approximately a 7:3 ratio.^20^ A flowchart of the inclusion criteria for liver cancer patients' images is shown in Figure 1. The detailed distribution of these lesion types within the dataset is presented in Table 1.

**TABLE 1:** The detailed distribution of three types of tumors in our dataset.

	Tumor type					
	HCC	ICC	MET	Cyst	HEM	Total
Training set	263	65	47	0	0	375
Test set	109	27	21	0	0	157
External validation set	17	5	7	8	8	45

Abbreviations: HCC, hepatocellular carcinoma; HEM, hemangiomas; ICC, intrahepatic cholangiocarcinoma; MET, metastatic liver cancer.

Additionally, we collected an external validation dataset comprising 52 patients with focal liver lesions from Hospital B. These lesions include not only malignant tumors such as HCC, ICC, and MET, but also benign lesions such as cysts and hemangiomas (HEM). All patients met the same inclusion and exclusion criteria as the internal dataset, seven patients were excluded. The CT scans were acquired during the portal venous phase using Canon CT scanners, with an in‐plane resolution of 512 × 512 pixels and a slice thickness of 2 mm. This external dataset was used to evaluate the generalization performance of our proposed method across different institutions and imaging devices. The development flow chart and detailed information of the external validation dataset are also presented in Figure 1 and Table 1.

### 2.2 Architecture overview

**FIGURE 2:**
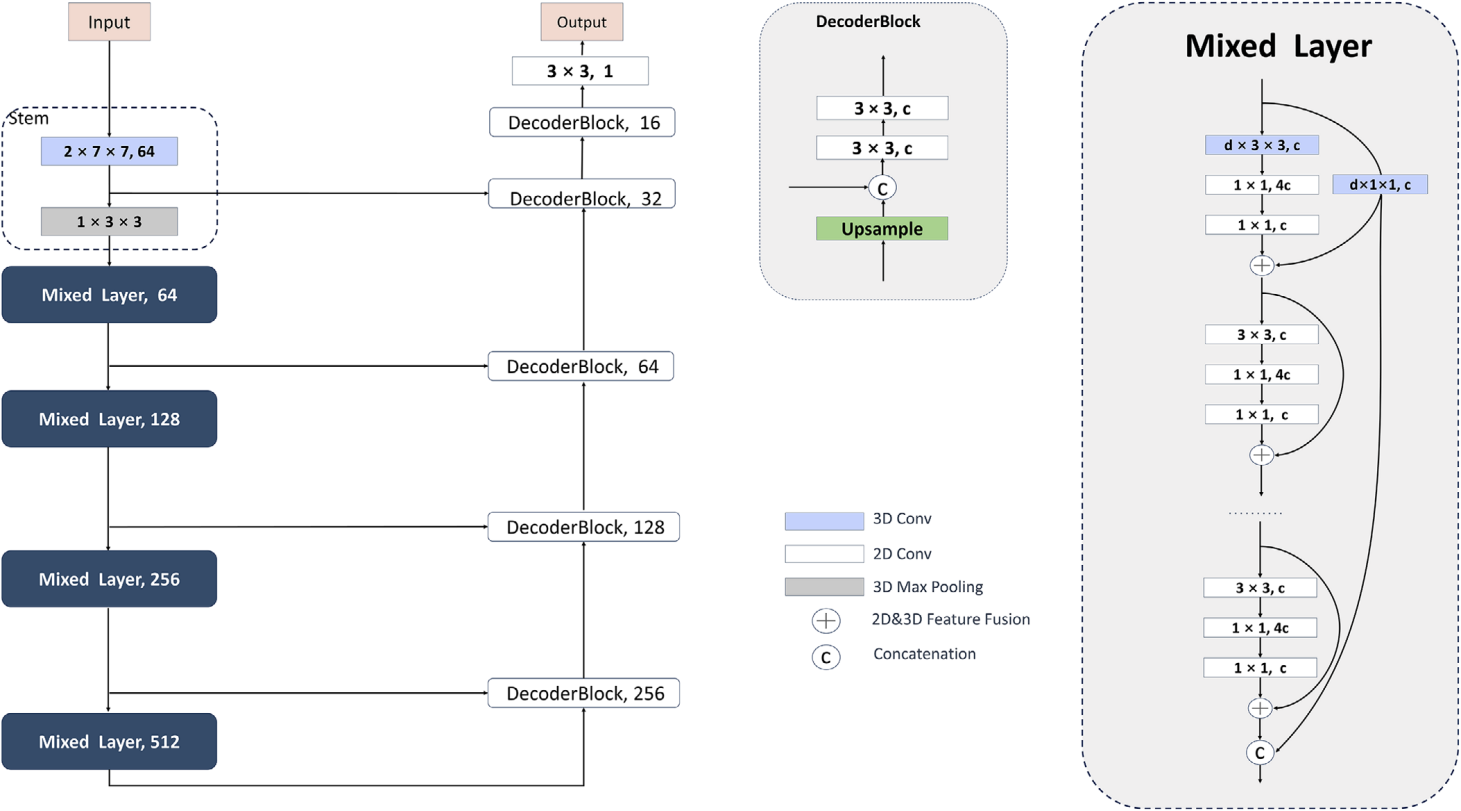
Overview of the Mixed U‐Net for automatic liver tumor segmentation. Parameter *d* represents the size of the depth dimension of the convolutional kernel, and *c* represents the number of output channels of the convolutional layer.

The architecture of the model is based on U‐Net, consisting of an encoder and a decoder. The network takes a stack of adjacent slices as input and outputs segmentation predictions for the central slice. Specifically, seven adjacent slices are stacked along the channel dimension, forming a seven‐channel input tensor. The encoder is based on the 2D ResNet^21^ architecture and is enhanced by incorporating 3D convolutional layers. This modification allows the network to effectively extract z‐axis features while only slightly increasing the number of parameters. The stem of the encoder consists of 3D convolutional layers and 3D max‐pooling layers. Each mixed layer is composed of multiple blocks stacked together. In the decoder part, each DecoderBlock consists of one nearest upsampling layer followed by two convolutional layers. The final layer in the decoder is a 3 × 3 2D convolutional layer, serving as the segmentation head to output the final segmentation results. The overall architecture of the network is illustrated in Figure 2.

### 2.3 Mixed layer

In the extraction of z‐axis features, 3D convolutional layers have a natural advantage. The “sliding window” strategy of convolutional layers is inherent in visual processing, and their inductive bias makes them effectively applicable in the field of computer vision.^22^ To construct an economical and lightweight model compared to 3D networks, we introduced a small number of 3D convolutions into the 2D network, leveraging the natural advantage of 3D convolution. 2D convolutional layers lead to the early fusion of information from all slices. Therefore, 3D convolutional layers should be positioned before 2D convolutional layers to extract z‐axis information, which results in the limitation of 3D convolutions to low‐level features in the network. To overcome this issue, this study employed residual connections and skip connections, this enables the network to reuse low‐level 3D features, thereby achieving finer‐grained feature extraction along the z‐axis. In the design of the first block in each mixed layer, a 3D convolutional layer was applied to process the 3D feature map of size N × C × D × H × W. This avoids the direct fusion of features from individual slices. The 3D convolutional layer performs downsampling, with a kernel size of d × 3 × 3 and corresponding strides of (1, 1, 1) or (1, 2, 2). Here, the parameter *d* was set to 2 or 3 to control the granularity of feature extraction along the depth dimension. Specifically, parameter *d* controls the size of the 3D convolutional kernel along the depth (z) axis, determining the number of adjacent slices used for feature integration. A larger *d* enables the model to capture broader spatial context across slices, while a smaller *d* focuses on finer‐grained, localized features within a narrower z‐axis range. After the 3D convolutional layer, two 1 × 1 2D convolutional layers are used to extract global features, which are then fused with the 3D local feature maps passed through the 3D residual connection. The global 2D feature maps used for fusion have the same number of channels, width, and height as the 3D local feature maps. They are expanded in the depth dimension by replication, and then directly added to the 3D local feature maps to achieve feature fusion. A block ultimately outputs a 3D feature map. To use the 3D feature maps as input for 2D convolution, the 3D feature maps need to be reshaped into feature maps of size N × C' × H × W, where C' = C × D. The same approach is applied to the skip connections of the U‐Net. If we referred to the bottleneck^21^ design, where the output channels of the block are four times wider than the input channels, the reshaping method mentioned above would result in an excessively large number of channels for the feature maps input to the 2D convolutional layers, which would lead to a significant increase in the number of parameters of both the encoder and decoder. To reduce the number of parameters and align with the number of channels in the feature maps after the 3D convolutional layer, we applied dimension expansion in the hidden convolutional layers instead, with an expansion rate of 4. Each layer embeds a 3D convolutional layer only in the first block, while subsequent blocks use 2D convolution to reduce the number of parameters and memory requirements. For the final output of each mixed layer, the outputs of the last block and the first residual connection are concatenated along the channel dimension. This skip connection compensates for long‐range information loss and enhances feature correlation before the next 3D convolutional layer.

### 2.4 Loss functions

For medical image lesion segmentation tasks, non‐lesion voxels in medical images are often far more numerous than lesion voxels. This can lead to biased segmentation results, which favor predictions of non‐lesion tissues and result in low sensitivity (Sen). In computer‐aided diagnosis or clinical decision‐support systems, false negatives are less tolerable than false positives, and high Sen is a key feature of automated detection systems.^23^ Tversky loss^23^ improves upon Dice loss^24^ by achieving a better balance between false positives and false negatives.

When training solely with Tversky loss for non‐lesion slices, where the ground truth values are all 0, it can lead to the gradient being 0. Slices without lesions cannot influence parameter updates in the model. For a complete abdominal CT volume of a patient, non‐lesion slices often constitute the majority of the total slices. The model needs to have the capability to recognize slices without lesions. Therefore, we introduced binary cross‐entropy (BCE) loss to encourage the model's learning of non‐lesion slices.

The complete loss function used in this paper is comprised of:

$$L=L_{\mathrm{Tversky}}+\lambda L_{\mathrm{BCE}}$$

The parameter *λ* is used to control the impact of BCE loss.

### 2.5 Implementation

We stacked seven adjacent slices as the input of the model, which provided the optimal balance between capturing sufficient z‐axis context and maintaining computational efficiency. For data augmentations, we employed simple data augmentation techniques: random rotation of slices within 30°, normalization with mean and standard deviation. For the configuration of the number of channels in each layer, the channel number of the encoder's Stem was set to 64, each Mixed Layer channel was set as [64, 128, 256, 512], a common design pattern that progressively expands feature channels while downsampling spatial dimensions, balancing representational capacity and computational cost., and each DecoderBlock was set as [256, 128, 64, 32, 16]. For the number of blocks in the mixed layer, we set it as [2, 2, 2, 2], which has a lower number of parameters while maintaining high segmentation performance and remains consistent with ResNet18 for ablation studies. We also provided a deeper Mixed U‐Net, with the number of blocks set as [9, 12, 8, 3], which has a higher number of parameters and achieves a higher Dice Score. *d* in each 3D convolution layer was set to [2, 2, 2, 3]—to perform fine‐grained feature extraction in early layers while capturing broader contextual information in deeper layers. *α* and *β* in Tversky loss were respectively set to 0.3 and 0.7 to emphasize recall over precision, as established in.^23^ The weight *λ* was set to 0.6 to effectively balance gradient contributions from both lesion and non‐lesion slices. The model was trained using the Adam optimizer with an initial learning rate of 2 × 10^−4^. It employed a cosine annealing scheduler with restarts, where the minimum learning rate was set to 1 × 10^−6^. The number of iterations for the first restart was set to 300. All models were trained on a server with NVIDIA RTX 3090 GPUs using PyTorch. To ensure fair comparison, each model was trained until full convergence on the same data splits. 2D and 2.5D models were trained for 450 epochs, while more complex 3D models required 1000 epochs. Batch sizes were set to the maximum feasible value per GPU to fully utilize available memory. The nnU‐Net^25^, ^26^ framework automatically configured its own hyperparameters based on dataset properties and hardware constraints. A complete breakdown of parameters, batch sizes, epoch counts, total training time and the average inference time per case for all models is provided in Table 2 of the results section.

**TABLE 2:** Model training and inference parameters.

		Parameters	Batch size	Epochs	Training time (h)	Inference time (ms)
2D	U‐Net	32.5 M	24	450	31	656
	UperNet	60.1 M	12	450	169	2471
	DeepLabV3+	37.0 M	24	450	50	745
	Segformer	44.6 M	16	450	101	1254
3D	U‐Net	73.2 M	2	1000	67	1515
	Swin UNETR	62.2 M	1	1000	183	6127
	nnU‐Net	30.5 M	2	1000	24	1796
	nnU‐Net ResEnc M	101 M	2	1000	30	2375
2D and 3D	AH‐Net	27.1 M	2	450	81	1535
	Mixed U‐Net	29.6 M	16	450	49	1178
	Deeper Mixed U‐Net	56.6 M	12	450	97	2133

### 2.6 Statistical analysis

We used common metrics to quantitatively analyze the model performance, including Sen, specificity (Spe), Dice similarity coefficient (per case Dice), volumetric overlap error (VOE), and relative volume difference (RVD). Ninety‐five percent confidence intervals (CI) were employed for estimate the ranges of these metrics. Dice was taken as the primary reference metric.

### 2.7 Clinical visual qualitative assessment

To rigorously evaluate the clinical relevance and practical utility of the segmentation results, a formal qualitative assessment was conducted by an experienced radiologist. The assessor was blinded to the model's predictions during the evaluation. The entire internal test set was subjected to this assessment. The radiologist reviewed the model's predictions slice‐by‐slice for every case. The assessment was based on the following binary criteria: Adequate—The segmentation result was considered sufficient for potential clinical application, such as treatment planning. A prediction was rated as Adequate if it captured the primary lesion without major omission, and the segmentation boundaries were anatomically plausible. Minor inaccuracies, such as a small degree of false positive or false negative segmentation at the lesion periphery, were permitted. The key criterion was that any such inaccuracies did not alter the overall clinical interpretation of the lesion's size, location, number, or relationship to critical anatomical structures, and thus would not compromise its value in clinical decision‐support. Inadequate—The segmentation result was deemed unfit for clinical use. This rating was assigned if there were severe defects that did not align with the above descriptions of an Adequate segmentation. The overall clinical applicability rate was calculated as the proportion of cases rated as Adequate to the total number of cases evaluated.

## 3 RESULTS

### 3.1 Comparison with popular methods

We compared Mixed U‐Net with several popular image segmentation networks, including convolutional‐based networks and transformer‐based networks. The experimental results are shown in Table 3. For 2D models: U‐Net, UperNet^27^ with ConvNeXt^22^ as the backbone, DeepLabV3+ with Xception^28^ as backbone, and Segformer. For 3D models: Swin UNETR,^29^ 3D U‐Net, nnU‐Net, ^25^ and nnU‐Net with residual encoder.^26^ For both 2D and 3D models, AH‐Net^30^ is a 3D Anisotropic Hybrid Network which stacks slices to pre‐train a 2.5D network with 2D ResNet50 as the encoder on ImageNet, and the network then extracts and transfers parameters of pre‐training 2D encoder to the corresponding 3D encoder layers of the 3D AH‐Net. Our proposed Mixed U‐Net with two different configurations achieved the best results, which both outperformed other popular methods in segmentation performance. The deeper network reached 84.51% sensitivity (95% CI: 84.37%–84.54%), 99.910% specificity (95% CI: 99.910%–99.911%), 81.54% Dice (95% CI: 81.45%–81.62%), 28.41% VOE (95% CI: 28.33%–28.52%), and 0.1196 RVD (95% CI: 0.1140–0.1203).

**TABLE 3:** Comparison of the mix 3D U‐Net with other popular methods.

		Backbone	Parameters	Sen	Spe	Dice	VOE	RVD
2D	U‐Net	Resnet50	32.5 M	81.13	99.903	78.04	32.70	0.2125
	UperNet	ConvNeXt	60.1 M	76.21	99.926	75.78	35.16	0.0930
	DeepLabV3+	Xception	37.0 M	76.57	99.930	77.21	33.32	0.0255
	Segformer		44.6 M	84.01	99.895	78.41	32.49	0.2529
3D	U‐net	Resnet50	73.2 M	76.52	99.921	76.63	32.85	−0.0196
	Swin UNETR		62.2 M	79.08	99.854	75.14	35.01	0.1940
	nnU‐Net		30.5 M	77.78	99.948	79.49	30.00	−0.0444
	nnU‐Net ResEnc M		101 M	78.06	99.951	80.70	28.65	−0.1213
2D and 3D	AH‐Net		27.1 M	82.26	99.901	80.20	29.97	0.0530
	Mixed U‐Net		29.6 M	82.38	99.922	81.12	28.98	0.0999
	Deeper Mixed U‐Net		56.6 M	84.51	99.910	81.54	28.41	0.1196

Abbreviations: Sen, sensitivity; Spe, specificity; RVD, relative volume difference; VOE, volumetric overlap error.

**FIGURE 3:**
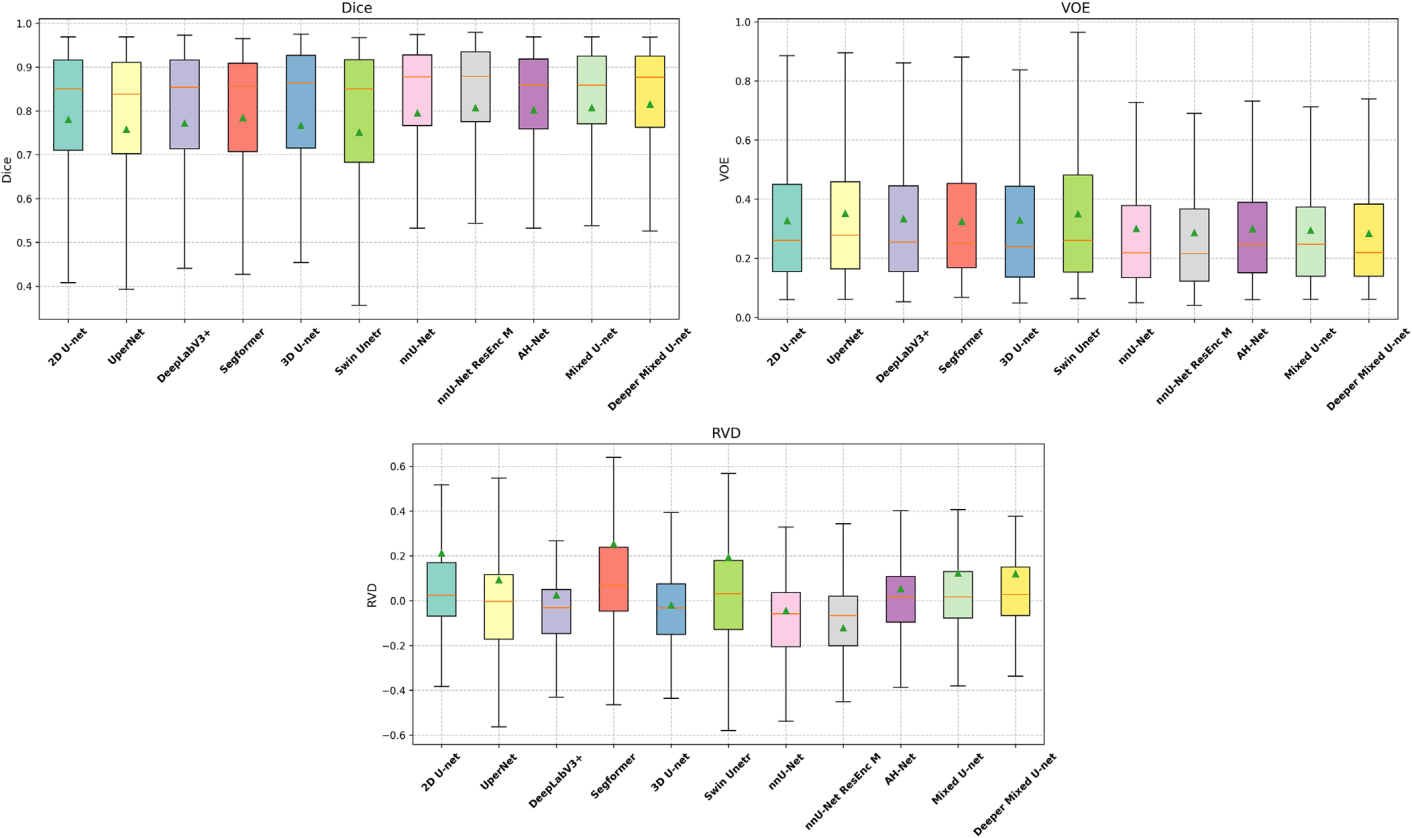
Box plots of Dice, volumetric overlap error (VOE), and relative volume difference (RVD) on the internal test set, with green triangles indicating the mean values.

As illustrated in Figure 3, the first quartile Q1 (lower bound of the box) of Dice and the third quartile Q3 (upper bound of the box) of VOE provide insight into the performance on the most challenging cases, since higher Dice and lower VOE represent better segmentation accuracy. The higher Q1 of Dice and lower Q3 of VOE for our model indicate that it effectively improves segmentation performance on difficult cases compared with other models. The median RVD of our model was closer to zero than that of the others, while the interquartile range is more symmetrically distributed around the median, suggesting that our model achieves more balanced predictions.

### 3.2 Ablation analysis of mixed U‐Net

To conduct a quantitative ablation analysis of the performance of Mixed U‐Net, we compared it with 2D, 3D, and 2.5D models based on the baseline U‐Net architecture. For experimental settings, the backbone of the encoder part for both 2D and 3D U‐Net utilized the 2D and 3D ResNet18 architectures, respectively. Due to memory constraints, 3D models were fed with 3D patches of size 192 × 192 × 32 as input. The 2.5D model stacked adjacent seven slices as input, different types of liver cancer consistent with the Mixed U‐Net. To assess the impact of parameter, increase on the models, we also trained a 2.5D ResNet50 model for comparison. The specific results of the ablation experiments are shown in Table 4. The proposed Mixed U‐net balances parameter efficiency and segmentation performance, with 82.38% sensitivity (95% CI: 82.26%–82.45%), 99.922% specificity (95% CI: 99.922%–99.923%), 81.12% Dice (95% CI: 81.04%–81.21%), 28.98% VOE (95% CI: 28.88%–29.08%), and 0.0999 RVD (95% CI: 0.0941–0.1035).

**TABLE 4:** Results of the ablation study.

	Backbone	Parameters	Sen	Spe	Dice	VOE	RVD
2D U‐Net	Resnet18	14.3 M	79.03	99.909	76.29	34.46	0.1975
3D U‐Net	Resnet18	42.6 M	77.60	99.881	74.15	36.09	0.3744
2.5D U‐Net	Resnet18	14.3 M	77.38	99.926	77.68	32.65	0.0429
2.5D U‐Net	Resnet50	32.5 M	81.08	99.923	79.91	30.09	0.0342
**Mixed U‐Net**		29.6 M	82.38	99.922	**81.12**	28.98	0.0999
2.5D U‐Net	Resnet101	51.5 M	81.36	99.913	80.22	30.05	0.0545
**Deeper Mixed U‐Net**		56.6 M	84.51	99.910	**81.54**	28.41	0.1196

Abbreviations: Sen, sensitivity; Spe, specificity; RVD, relative volume difference; VOE, volumetric overlap error.

### 3.3 The impact of different loss functions

We experimented with using Dice loss only, Tversky loss only, and the combination of Tversky loss and BCE loss separately in Mixed U‐net. The results are shown in Table 5. Our loss function achieved the best performance. In contrast to Dice loss, Tversky loss led to a notable increase in Sen and Dice score as expected, BCE loss further improved the Dice score with a slight decrease in Sen. To determine the optimal value for the loss weight *λ*, we conducted a Sen analysis by varying *λ* while keeping all other parameters constant. The results, summarized in Table 5, show that the model performance is robust across a range of *λ* values, with the optimal Dice score achieved at *λ* = 0.6. This validates our empirical choice.

**TABLE 5:** Comparison of the different loss functions.

	Sen	Spe	Dice	VOE	RVD
Dice loss	78.93	99.938	79.37	30.73	0.0360
Tversky loss	83.19	99.900	80.25	29.91	0.1006
Tversky loss + BCE loss (*λ* = 0.2)	81.82	99.934	80.90	29.14	0.0471
Tversky loss + BCE loss (*λ* = 1.0)	81.52	99.931	81.08	28.89	0.0173
Tversky loss + BCE loss (*λ* = 0.6)	82.38	99.922	81.12	28.98	0.0999

Abbreviations: Sen, sensitivity; Spe, specificity; RVD, relative volume difference; VOE, volumetric overlap error.

### 3.4 Testing different number of input slices

Stacking different numbers of slices as input to Mixed U‐net allows us to test the effects of varying slice numbers and the model's capacity for handling different numbers of slices. The selection of the optimal number of slices may also depend on the dataset itself. For example, different types of medical imaging segmentation tasks or even different types of liver cancer may have varying impacts on the optimal slice number. The model with seven slices as input has the highest Dice Score, three‐slice and five‐slice exhibit relatively lower results, there is a gradual decrease in performance from 7 to 11. The results are shown in Table 6. We computed the Structural Similarity Index (SSIM) between the first and last slices for input stacks ranging from 3 to 11 slices. As shown in Table 6, the SSIM values decreased progressively as the number of slices increased, indicating reduced similarity between distant slices.

**TABLE 6:** Result of stacking different numbers of slices as input.

	SSIM	Sen	Spe	Dice	VOE	RVD
Three‐slice	75.85	82.82	99.915	80.40	29.84	0.1573
Five‐slice	70.10	83.75	99.907	80.43	29.79	0.2147
Seven‐slice	67.39	82.38	99.922	81.12	28.98	0.0999
Nine‐slice	65.66	81.04	99.918	80.03	30.17	0.1231
Eleven‐slice	64.44	80.44	99.923	79.69	30.51	0.0394

Abbreviations: Sen, sensitivity; Spe, specificity; SSIM, structural similarity index; RVD, relative volume difference; VOE, volumetric overlap error.

### 3.5 Visualization of segmentation results and clinical assessment

**FIGURE 4:**
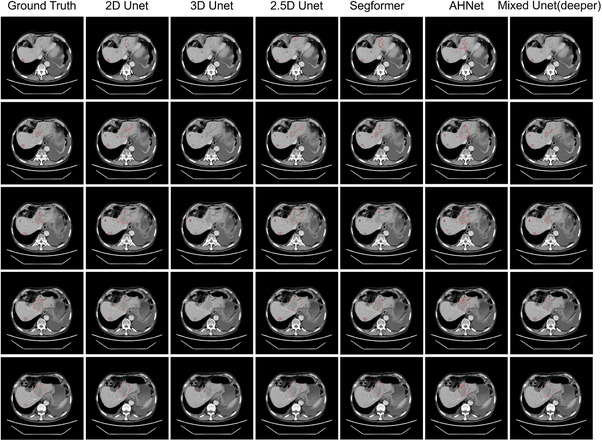
Visualization results. Mixed U‐Net accurately distinguishes tumor from vessels in a challenging case.

We further compared the visualization segmentation results on 2D U‐Net (ResNet50), 2.5D U‐Net (ResNet50), 3D U‐Net (ResNet50), Segformer, AH‐Net, and Deeper Mixed U‐Net. We present the prediction results of adjacent slices from the same case. Due to the influence of blood vessels, this case is relatively challenging to segment. Figure 4 illustrates the prediction results of different models. Our model outperformed others in accurately distinguishing between lesion tissue and vascular tissue, which achieved the best segmentation results. The 2D U‐Net and 2.5D U‐Net struggle to make effective distinctions, the 2.5D U‐Net segments both lesions and the vascular tissues adjacent to the lesions. The 3D U‐Net yields the poorest results. AH‐Net exhibits confusion regarding the information of slices, as it produced identical segmentation for different slices.

**FIGURE 5:**
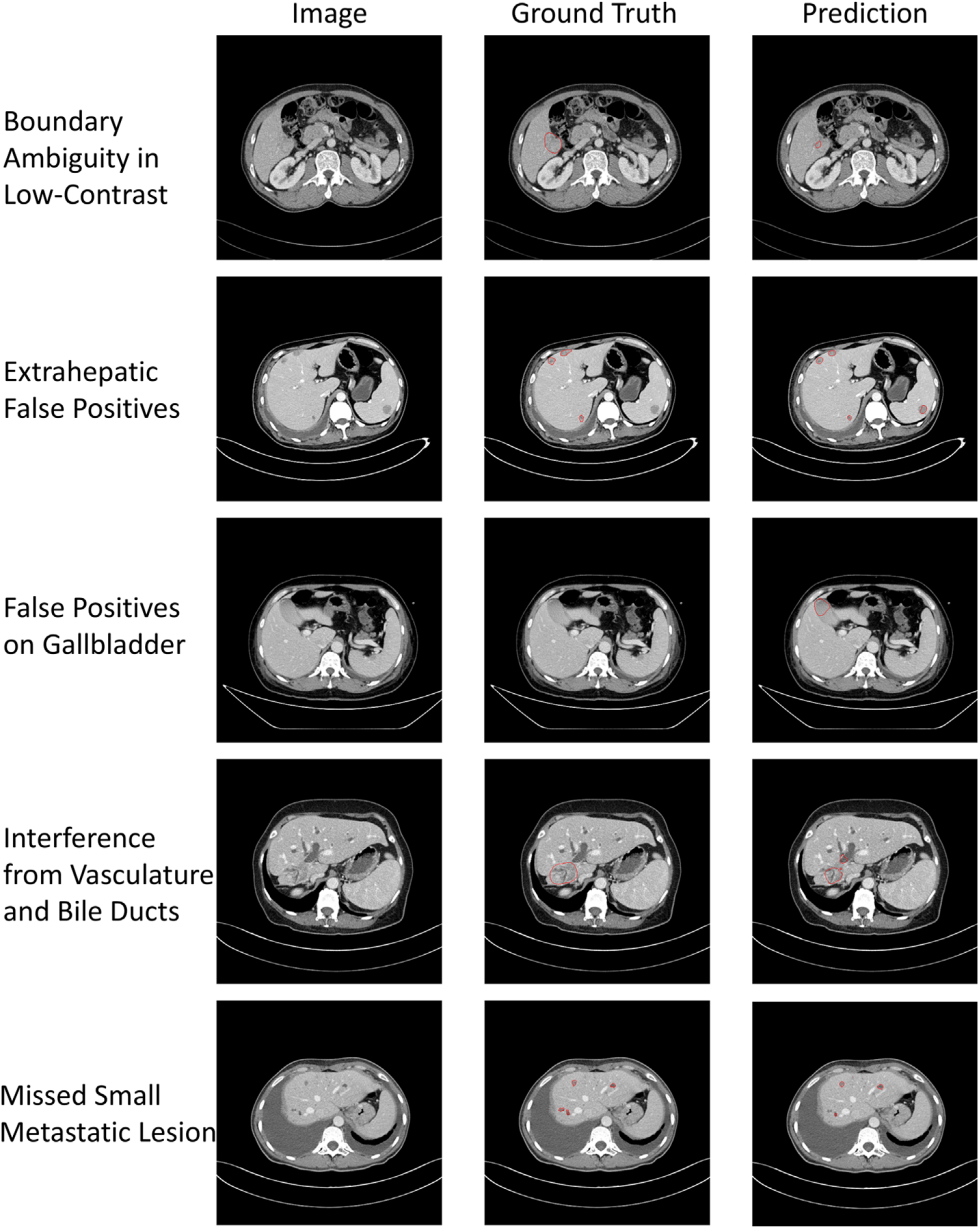
Representative examples of five failure modes.

A specialist radiologist performed a qualitative assessment of the entire test set, comprising 157 cases, evaluating the model's predictions on a slice‐by‐slice basis. The clinical applicability rate was 83.44%, with a certain number of cases deemed Adequate. Twenty‐five cases in total were rated as Inadequate, accounting for 15.9% of the test set. Analysis of these cases revealed five common causes of segmentation failure. The most frequent issue was low contrast between the tumor and surrounding liver tissue, which resulted in segmentation errors in 11 cases. Other errors included the misclassification of hypodense areas in extrahepatic organs (e.g., the spleen) as tumors in two cases. Additionally, the model incorrectly segmented the gallbladder in three cases. Confusion with vascular structures or dilated bile ducts caused boundary ambiguity or missegmentation in five cases. Finally, the model failed to detect small metastatic tumors in five cases. Representative examples of these failure modes can be found in Figure 5.

### 3.6 External validation

**FIGURE 6:**
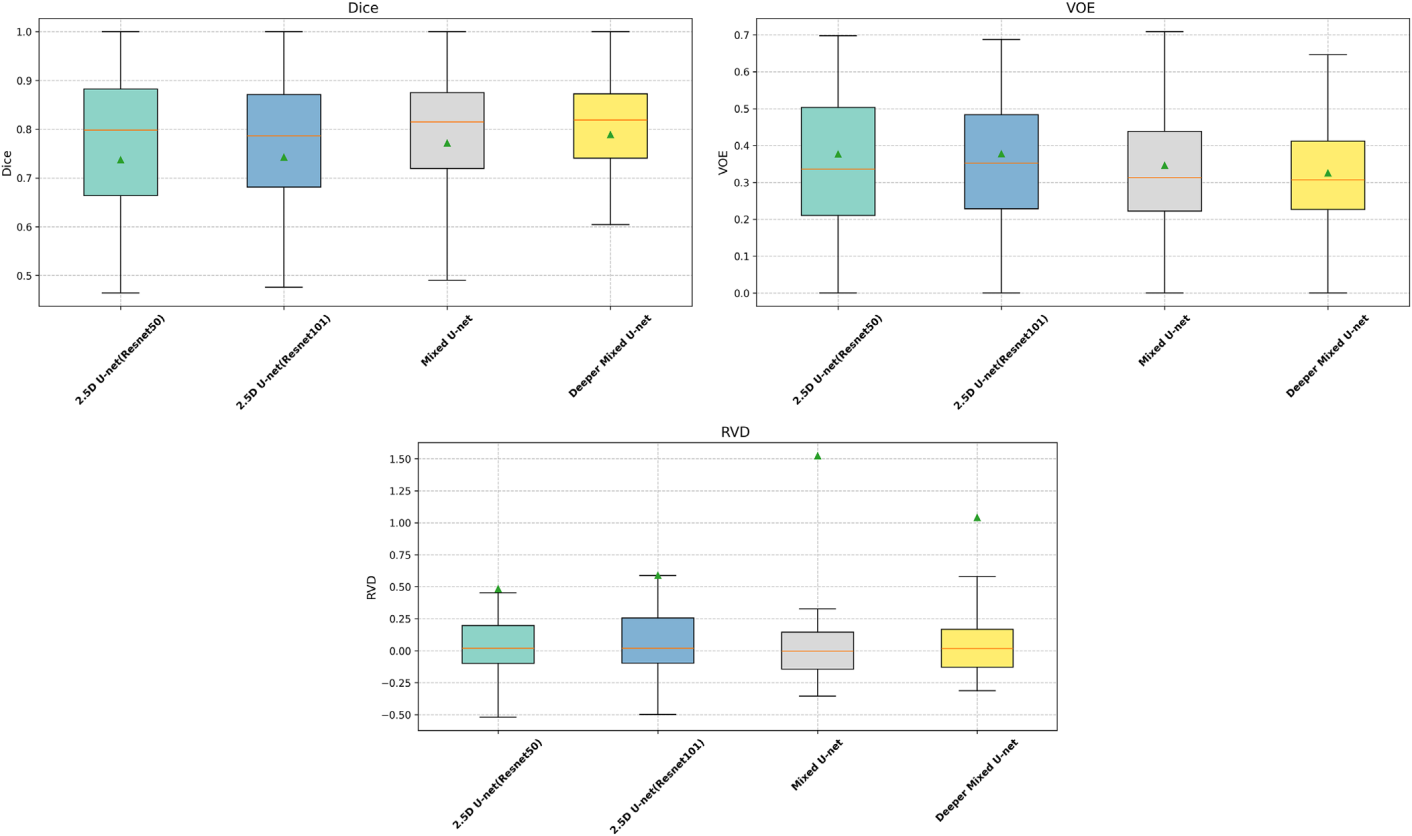
Box plots of Dice, volumetric overlap error (VOE), and relative volume difference (RVD) on the external validation set, with green triangles indicating the mean values.

To evaluate the generalization ability of our proposed model, we conducted external validation using an independent dataset consisting of 45 liver cancer patients collected from Hospital B. As shown in Table 7, our Mixed U‐Net demonstrated strong robustness and generalization performance when applied to data from a different institution and CT scanner vendor (Canon). Compared to the internal test results, several baseline models exhibited a noticeable drop in performance on the external dataset. In contrast, Mixed U‐Net maintained competitive segmentation accuracy, while the Deeper Mixed U‐Net achieved the best overall performance, with a Dice coefficient of 78.92% (95% CI: 78.79%–79.08%), Sen of 83.06% (95% CI: 82.91%–83.17%), Spe of 99.943% (95% CI: 99.943%–99.945%), VOE of 32.55% (95% CI: 32.38%–32.72%), and RVD of 1.0417 (95% CI: 0.9470–1.0621). Notably, as the number of parameters increased, the Deeper Mixed U‐Net showed clear improvements in both Sen and Dice score, confirming that the deeper architecture can better capture inter‐slice contextual features and adapt to variations across datasets. The boxplots of Dice, VOE, and RVD on the external validation set are shown in Figure 6. The shorter interquartile range observed in the boxplot further also indicates the robustness of our method.

**TABLE 7:** Results of the external validation.

	Parameter	Sen	Spe	Dice	VOE	RVD
2.5D U‐Net (Resnet50)	32.5 M	79.45	99.951	73.75	37.64	0.4835
2.5D U‐Net (Resnet101)	51.5 M	80.24	99.948	74.25	37.32	0.5882
Mixed U‐Net	29.6 M	80.01	99.951	77.16	34.65	1.5231
Deeper Mixed U‐Net	56.6 M	83.06	99.943	78.92	32.55	1.0417

Abbreviations: Sen, sensitivity; Spe, specificity; RVD, relative volume difference; VOE, volumetric overlap error.

## 4 DISCUSSION

To address clinical challenges in liver tumor segmentation, including unclear boundaries, vessel and duct misclassification, and wide lesion variability, we propose a hybrid 2D and 3D model by integrating 2D and 3D convolutions. This model achieved optimal performance with relatively low computational cost, demonstrating that embedding 3D convolutional layers into 2D networks is an effective strategy for improving the performance of medical image segmentation. The Mixed U‐Net achieved significant improvements in segmentation performance than 2D Networks for the lack of contextual information along the z‐axis of the slices, even 2D networks like U‐Net, UperNet, and DeepLabV3+ with a larger number of parameters fail to attain higher performance. This is attributed to our improvements in the block of mixed‐layer and the strategy of introducing only a small number of 3D convolutional layers. In contrast to the 2.5D U‐Net with backbone of ResNet18, our Mixed U‐Net also achieved notable improvement. Taking into account the impact of parameter increment, our model similarly attained a higher Dice score than 2.5D U‐Net with backbone of ResNet50, even with a lower number of parameters. All 3D models exhibited relatively poor performance. We believe this is primarily due to memory constraints, which limit their performance and prevent them from accessing sufficient global information across the complete volume. Models based on transformers can capture global features, while convolutional neural networks are limited by their local receptive fields in capturing long‐range contextual features. The transformer‐based techniques in the field of medical imaging have witnessed exponential growth, especially since the introduction of Vision Transformer.^31^ Our convolutional‐based model outperformed transformer‐based models like Segformer and Swin UNETR based on the Swin Transformer^32^ encoder on our dataset. Additionally, when training Segformer using the 2.5D approach, the model failed to converge. Our model effectively reduced computational time compared to full 3D networks while being slower than 2D networks, as expected. nnU‐Net's efficient engineering and decoupled preprocessing contributed to its faster training time. Our approach differs from nnU‐Net in that the latter employs a more engineering‐driven pipeline, equipped with powerful data processing routines and extensive data augmentation strategies during training, which further enhance its segmentation performance.

Considering the inherent imbalance between lesion and non‐lesion voxels in medical images, as well as the practical requirements during model inference, we introduced the Tversky loss and BCE loss instead of Dice loss which is commonly used in image segmentation tasks. Our loss resulted in higher Dice scores for the Mixed U‐Net. The Tversky loss led to a relatively significant improvement in performance, which effectively addresses the imbalance between the lesion and non‐lesion voxels. The BCE loss further improved the Dice score, this demonstrates that no‐lesion slices have a substantial impact on the segmentation performance, especially when predicting the entire CT volume of a patient.

In slices with focal liver lesions, adjacent slices have a higher correlation compared to slices further apart, which is sufficient for making accurate predictions on slices with lesions. So, it is unnecessary to input all slices into the model, the information fusion of slices is not conducive to making accurate predictions for small lesions. When the number of input slices ranges from 3 to 7, the model performance increases. However, for stacks of 9 or 11 slices, the segmentation performance decreases. This aligns with our observations of the CT data of our dataset. When the number of adjacent slices exceeds approximately 7, the inter‐slice correlation decreases significantly in some patients. The SSIM analysis revealed that increasing the number of input slices led to gradually decreasing similarity between distant slices. The seven‐slice configuration achieved an optimal balance between incorporating multi‐slice contextual information and maintaining feature correlation. Beyond seven slices, additional slices provided diminishing relevant information while potentially introducing more noise, resulting in segmentation performance degradation. The selection of the optimal number of slices may also depend on the dataset itself.

In clinical practice, liver vasculature, bile duct dilatation, and artifacts caused by respiratory motion can all lead to erroneous segmentation. Radiologists rely on sufficient contextual information from multiple slices to make accurate segmentations. Benefiting from the embedding of 3D convolutional layers and the fine‐grained z‐axis feature extraction approach, the Mixed U‐Net leverages the inter‐slice contextual information to accurately segment both lesion and non‐lesion tissues in difficult cases, whereas the 2.5D U‐Net, with a similar structure and the same input, exhibits poorer discriminative ability due to the early fusion of information. The 3D U‐Net, constrained by memory limitations, is often trained on small patches rather than full volumes. This limits its ability to capture broader contextual information, leading to inferior performance. Besides, the network design (stacking of max pooling layers) of AHNet also results in the early fusion of adjacent slice information, thereby leading to the confusion of inter‐slice features and making identical predictions for different slices. Our clinical qualitative evaluation further strengthens the practical value of our method. The high clinical applicability rate of 83.44% demonstrates that the improved Dice score of our Mixed U‐Net translates to clinically useful segmentations. Analysis of the Inadequate cases primarily revealed failures in low‐contrast scenarios and confusion with adjacent structures—challenges consistent with manual segmentation. This evaluation confirms that our model's performance extends beyond theoretical metrics to offer genuine clinical utility.

In an external validation study using data from another hospital, the Mixed U‐Net demonstrated significantly better segmentation performance than the traditional 2.5D U‐Net. This indicates that the proposed architecture can more effectively integrate contextual information across adjacent slices, enhancing the model's ability to recognize complex anatomical structures such as liver vasculature and bile ducts. Moreover, as the number of model parameters increased, the Mixed U‐Net showed improved feature representation and higher Dice scores on external data, while the performance of the 2.5D U‐Net remained relatively unchanged. These findings suggest that the Mixed U‐Net, by incorporating sparse 3D convolutional modules, has a stronger capacity to model spatial continuity and maintain segmentation stability across variable imaging conditions. Its robust performance on multi‐center data supports its clinical applicability, offering reliable, and generalizable results that are well‐suited for use in diverse clinical environments.

There are still some shortcomings awaiting exploration and improvement in the future. Regarding the fusion of 2D and 3D features, we only employed a coarse fusion strategy, which could be further refined to enhance the fusion of global and local features. Additionally, concerning our dataset, we did not address the issue of data imbalance caused by different types of lesions, which remains a problem to be solved and could further improve segmentation performance, and the exclusion of patients with prior treatments may limit the model's generalizability to real‐world clinical populations. Therefore, the inclusion of balanced distribution of lesion types and post‐treatment cases is necessary to enhance the size and diversity of the dataset. Furthermore, the variation in slice thickness (2 vs. 5 mm) was not normalized in this study. While our method demonstrated robustness to this variability, further investigation into anisotropic processing techniques represents an important direction for future work to better handle multi‐resolution clinical data.

Therapeutic methods such as radiofrequency ablation,^33^ thermal percutaneous ablation,^34^ surgical resection,^35^ and arterial embolization^36^ require precise segmentation of liver lesions.^3^ Manual lesion segmentation is a subjective assessment, our model can facilitate standardization in the clinical treatment processes.

## 5 CONCLUSIONS

This paper proposed a Mixed U‐Net, the design of network architecture integrates both 2D and 3D convolutions, we utilize residual connections and skip connections to assist the embedding of 3D convolutions into the 2D convolutional network and achieve fine‐grained feature extraction. Our method simultaneously considers computational cost and segmentation performance, which outperform some other popular 2D, 2.5D, and 3D methods. It also indicates that the network architecture combining 2D and 3D convolutions is a viable way compared to purely 2D or 3D networks, which has a positive contribution to feature extraction and network performance.

## AUTHOR CONTRIBUTIONS

Conceptualization, Feifei Gao. Methodology, Zheming Hu. Software, Zheming Hu. Validation, Feifei Gao and Zheming Hu; formal analysis, Zheming Hu. Investigation, Feifei Gao. Resources, Jiajun Xian and Wei Lu. Data curation, Feifei Gao and Wei Lu. Writing—original draft preparation, Feifei Gao and Zheming Hu. Writing—review and editing, Jiajun Xian and Wei Lu. Visualization, Zheming Hu. Supervision, Jiajun Xian; project administration, Wei Lu. Funding acquisition, Wei Lu. All authors have read and agreed to the published version of the manuscript.

## CONFLICT OF INTEREST STATEMENT

The authors of this manuscript declare no relationships with any companies, whose products or services may be related to the subject matter of the article.

## ETHICAL APPROVAL STATEMENT

Ethics committee approval was granted by the ethics review board of West China Hospital of Sichuan University (Ethical Approval No. 2024–424).

## INFORMED CONSENT

Written informed consent was waived by the Institutional Review Board.

## References

[1] Sung H , Ferlay J , Siegel RL , et al. Global cancer statistics 2020: GLOBOCAN estimates of incidence and mortality worldwide for 36 cancers in 185 countries. CA Cancer J Clin. 2021;71(3):209–249. 33538338 10.3322/caac.21660

[2] Wang ZG , He ZY , Chen YY , Gao H , Du XL . Incidence and survival outcomes of secondary liver cancer: a surveillance epidemiology and end results database analysis. Transl Cancer Res. 2021;10(3):1273. doi:10.21037/tcr‐20‐3319 35116454 10.21037/tcr-20-3319 PMC8797763

[3] Bilic P , Christ P , Li HB , et al. The liver tumor segmentation benchmark (LiTS). Med Image Anal. 2023;84:102680. doi:10.1016/j.media.2022.102680 36481607 10.1016/j.media.2022.102680 PMC10631490

[4] Alvaro D , Gores GJ , Walicki J , et al. EASL‐ILCA Clinical Practice Guidelines on the management of intrahepatic cholangiocarcinoma. J Hepatol. 2023;79(1):181–208. doi:10.1016/j.jhep.2023.03.010 37084797 10.1016/j.jhep.2023.03.010

[5] Schindl MJ , Redhead DN , Fearon KCH , Garden OJ , Wigmore SJ . The value of residual liver volume as a predictor of hepatic dysfunction and infection after major liver resection. Gut. 2005;54(2):289–296. doi:10.1136/gut.2004.046524 15647196 10.1136/gut.2004.046524 PMC1774834

[6] Wei Y , Yang M , Zhang M , et al. Focal liver lesion diagnosis with deep learning and multistage CT imaging. Nat Commun. 2024;15(1):7040. doi:10.1038/s41467‐024‐51260‐6 39147767 10.1038/s41467-024-51260-6 PMC11327344

[7] Yang M , Yang L , Zhang Q , et al. Deep learning‐based magnetic resonance imaging analysis for chronic cerebral hypoperfusion risk. Med Phys. 2024;51(8):5270–5282. doi:10.1002/mp.17237 38820428 10.1002/mp.17237

[8] Raman AG , Jones C , Weiss CR . Machine learning for hepatocellular carcinoma segmentation at MRI: radiology in training. Radiology. 2022;304(3):509–515. doi:10.1148/radiol.212386 35536132 10.1148/radiol.212386

[9] Zhang Y , Liao Q , Ding L , Zhang J . Bridging 2D and 3D segmentation networks for computation‐efficient volumetric medical image segmentation: an empirical study of 2.5D solutions. Comput Med Imaging Graph. 2022;99:102088. doi:10.1016/j.compmedimag.2022.102088 35780703 10.1016/j.compmedimag.2022.102088

[10] Long J , Shelhamer E , Darrell T . Fully convolutional networks for semantic segmentation. In: Proceedings of the IEEE Conference on Computer Vision and Pattern Recognition. Institute of Electrical and Electronics Engineers; 2015:3431–3440.

[11] Ronneberger O , Fischer P , Brox T . U‐Net: convolutional networks for biomedical image segmentation. In Medical Image Computing and Computer‐Assisted Intervention–MICCAI 2015: 18th International Conference, Munich, Germany, October 5–9, 2015, Proceedings, Part III 18. Springer International Publishing; 2015:234–241.

[12] Zhou Z , Rahman Siddiquee MM , Tajbakhsh N , Liang J U‐Net++: a nested U‐Net architecture for medical image segmentation. In: Deep Learning in Medical Image Analysis and Multimodal Larning for Clinical Decision Support: 4th International Workshop, DLMIA 2018, and 8th International Workshop, ML‐CDS 2018, Held in Conjunction with MICCAI 2018, Granada, Spain, September 20, 2018, Proceedings 4. Springer International Publishing; 2018:3–11. 10.1007/978-3-030-00889-5_1 PMC7329239 32613207

[13] Chen LC , Zhu Y , Papandreou G , Schroff F , Adam H Encoder‐decoder with atrous separable convolution for semantic image segmentation. In Proceedings of the European Conference on Computer Vision (ECCV). European Conference on Computer Vision; 2018:801–818.

[14] Dosovitskiy A , Beyer L , Kolesnikov A , et al. An image is worth 16 × 16 words: transformers for image recognition at scale. arXiv preprint. 2020. arXiv:2010.11929.

[15] Xie E , Wang W , Yu Z , Anandkumar A , Alvarez JM , Luo P . SegFormer: simple and efficient design for semantic segmentation with transformers. Adv Neural Inform Process Syst. 2021;34:12077–12090.

[16] Qu Y , Li X , Yan Z , et al. Surgical planning of pelvic tumor using multi‐view CNN with relation‐context representation learning. Med Image Anal. 2021;69:101954. doi:10.1016/j.media.2020.101954 33550006 10.1016/j.media.2020.101954

[17] Çiçek Ö , Abdulkadir A , Lienkamp SS , Brox T , Ronneberger O . 3D U‐Net: learning dense volumetric segmentation from sparse annotation. In Medical Image Computing and Computer‐Assisted Intervention–MICCAI 2016: 19th International Conference, Athens, Greece, October 17–21, 2016, Proceedings, Part II 19. Springer International Publishing; 2016:424–432.

[18] Yu Q , Xia Y , Xie L , Fishman EK , Yuille AL . Thickened 2D networks for efficient 3D medical image segmentation. arXiv preprint. 2019. arXiv:1904.01150.

[19] Francis IR , Cohan RH , McNulty NJ , et al. Multidetector CT of the liver and hepatic neoplasms: effect of multiphasic imaging on tumor conspicuity and vascular enhancement. Am J Roentgenol. 2003;180(5):1217–1224. doi:10.2214/ajr.180.5.1801217 12704026 10.2214/ajr.180.5.1801217

[20] Gholamy A , Kreinovich V , Kosheleva O . Why 70/30 or 80/20 relation between training and testing sets: a pedagogical explanation. 2018.

[21] He K , Zhang X , Ren S , Sun J . Deep residual learning for image recognition. In: Proceedings of the IEEE Conference on Computer Vision and Pattern Recognition. Institute of Electrical and Electronics Engineers; 2016:770–778.

[22] Liu Z , Mao H , Wu CY , Feichtenhofer C , Darrell T , Xie S . A convnet for the 2020s. In: Proceedings of the IEEE/CVF Conference on Computer Vision and Pattern Recognition. Institute of Electrical and Electronics Engineers; 2022:11976–11986.

[23] Salehi SSM , Erdogmus D , Gholipour A . Tversky loss function for image segmentation using 3D fully convolutional deep networks. International Workshop on Machine Learning in Medical Imaging. Springer International Publishing; 2017:379–387.

[24] Milletari F , Navab N , Ahmadi SA . V‐net: fully convolutional neural networks for volumetric medical image segmentation. In: 2016 Fourth International Conference on 3D Vision (3DV). Institute of Electrical and Electronics Engineers; 2016:565–571. doi:10.1109/3DV.2016.79

[25] Isensee F , Jaeger PF , Kohl SA , Petersen J , Maier‐Hein KH . nnU‐Net: a self‐configuring method for deep learning‐based biomedical image segmentation. Nat Methods. 2021;18(2):203–211. doi:10.1038/s41592‐020‐01008‐z 33288961 10.1038/s41592-020-01008-z

[26] Isensee F , Wald T , Ulrich C , et al. nnU‐Net revisited: a call for rigorous validation in 3D medical image segmentation. In: International Conference on Medical Image Computing and Computer‐Assisted Intervention. Springer Nature Switzerland; 2024:488–498.

[27] Xiao T , Liu Y , Zhou B , Jiang Y , Sun J . Unified perceptual parsing for scene understanding. In: Proceedings of the European Conference on Computer Vision (ECCV). European Conference on Computer Vision; 2018:418–434.

[28] Chollet F . Xception: deep learning with depthwise separable convolutions. In: Proceedings of the IEEE Conference on Computer Vision and Pattern Recognition. Institute of Electrical and Electronics Engineers; 2017:1251–1258.

[29] Hatamizadeh A , Nath V , Tang Y , Yang D , Roth HR , Xu D . Swin UNETR: swin transformers for semantic segmentation of brain tumors in MRI images. International MICCAI Brainlesion Workshop. Springer International Publishing; 2021:272–284.

[30] Liu S , Xu D , Zhou SK , et al. 3D anisotropic hybrid network: transferring convolutional features from 2D images to 3D anisotropic volumes. In Medical Image Computing and Computer Assisted Intervention–MICCAI 2018: 21st International Conference, Granada, Spain, September 16–20, 2018, Proceedings, Part II 11. Springer International Publishing; 2018:851–858.

[31] Shamshad F , Khan S , Zamir SW , et al. Transformers in medical imaging: a survey. Med Image Anal. 2023;88:102802. doi:10.1016/j.media.2023.102802 37315483 10.1016/j.media.2023.102802

[32] Liu Z , Lin Y , Cao Y , et al. Swin transformer: hierarchical vision transformer using shifted windows. In: Proceedings of the IEEE/CVF International Conference on Computer Vision. Institute of Electrical and Electronics Engineers; 2021:10012–10022.

[33] Shah DR , Green S , Elliot A , McGahan JP , Khatri VP . Current oncologic applications of radiofrequency ablation therapies. World J Gastrointest Oncol. 2013;5(4):71. doi:10.4251/wjgo.v5.i4.71 23671734 10.4251/wjgo.v5.i4.71 PMC3648666

[34] Shiina S , Sato K , Tateishi R , et al. Percutaneous ablation for hepatocellular carcinoma: comparison of various ablation techniques and surgery. Can J Gastroenterol Hepatol. 2018;2018(1):4756147. 29974040 10.1155/2018/4756147 PMC6008833

[35] Albain KS , Swann RS , Rusch VW , et al. Radiotherapy plus chemotherapy with or without surgical resection for stage III non‐small‐cell lung cancer: a phase III randomized controlled trial. Lancet. 2009;374(9687):379–386. doi:10.1016/S0140‐6736(09)60737‐6 19632716 10.1016/S0140-6736(09)60737-6 PMC4407808

[36] Virdis F , Reccia I , Di Saverio S , et al. Clinical outcomes of primary arterial embolization in severe hepatic trauma: a systematic review. Diagn Interv Imaging. 2019;100(2):65–75. doi:10.1016/j.diii.2018.10.004 30555019 10.1016/j.diii.2018.10.004

